# FOXC1 transcriptionally suppresses ABHD5 to inhibit the progression of renal cell carcinoma through AMPK/mTOR pathway

**DOI:** 10.1007/s10565-024-09899-w

**Published:** 2024-08-02

**Authors:** Jianfa Li, Shuangchen Chen, Jing Xiao, Jiayuan Ji, Chenchen Huang, Ge Shu

**Affiliations:** 1 Department of Urology, Beijing Friendship Hospital, Capital Medical University, Beijing, China; 2 Department of Urology, Peking University First Hospital, Beijing, China; 3 Shenzhen Key Laboratory of Male Reproductive Medicine and Genetics, Institute of Urology, Peking University Shenzhen Hospital, Shenzhen, Guangdong China; 4 Department of Urology, Shanghai General Hospital, Shanghai Jiao Tong University School of Medicine, Shanghai, China

**Keywords:** FOXC1, ABHD5, AMPK, mTOR, Renal cell carcinoma

## Abstract

**Background:**

Increased activity of the transcription factor FOXC1 leads to elevated transcription of target genes, ultimately facilitating the progression of various cancer types. However, there are currently no literature reports on the role of FOXC1 in renal cell carcinoma.

**Methods:**

By using RT-qPCR, immunohistochemistry and Western blotting, FOXC1 mRNA and protein expression was evaluated. Gain of function experiments were utilized to assess the proliferation and metastasis ability of cells. A nude mouse model was created for transplanting tumors and establishing a lung metastasis model to observe cell proliferation and spread in a living organism. Various techniques including biological analysis, CHIP assay, luciferase assay, RT-qRCR and Western blotting experiments were utilized to investigate how FOXC1 contributes to the transcription of ABHD5 on a molecular level. FOXC1 was assessed by Western blot for its impact on AMPK/mTOR signaling pathway.

**Results:**

FOXC1 is down-regulated in RCC, causing unfavorable prognosis of patients with RCC. Further experiments showed that forced FOXC1 expression significantly restrains RCC cell growth and cell metastasis. Mechanically, FOXC1 promotes the transcription of ABHD5 to activate AMPK signal pathway to inhibit mTOR signal pathway. Finally, knockdown of ABHD5 recovered the inhibitory role of FOXC1 overexpression induced cell growth and metastasis suppression.

**Conclusion:**

In general, our study demonstrates that FOXC1 exerts its tumor suppressor role by promoting ABHD5 transcription to regulating AMPK/mTOR signal pathway. FOXC1 could serve as both a diagnostic indicator and potential treatment focus for RCC.

**Supplementary Information:**

The online version contains supplementary material available at 10.1007/s10565-024-09899-w.

## Introduction

In 2020, there were 431,288 new renal cell carcinoma (RCC) cases and 179,368 deaths worldwide (Sung et al. 2021). RCC is too concealed to be discovered. Although most accidentally detected lesions are diagnosed as low-grade tumors, approximately 17% of RCC patients have distant metastases at first diagnosis (Siegel et al. 2014). Ten-years survival rate of earlier stage kidney cancer patients is 85% to96% (Shuch et al. 2015), and advanced RCC patients lost the opportunity become unsectable. However, the prognosis of advanced RCC patients is still dissatisfactory despite of immunotherapy and targeted therapy (Ciccarese et al. 2017; Posadas et al. 2017). What was worse, few diagnostic and prognostic biomarkers could be used in the clinical work. Therefore, it is important to investigate novel therapeutic target and signal pathway to fight against RCC.

The fokhead box (FOX) proteins containing a distinct DNA-binding FOX domain are characterized as evolutionarily conserved transcription factors (Katoh et al. 2013). FOX proteins have the ability to control transcriptional processes and are crucial in the development of embryos and organs, as well as in cell differentiation, growth, and programmed cell death (Hannenhalli and Kaestner 2009; Katoh and Katoh 2004; Pohl et al. 2005; Xu et al. 2021; Zeng et al. 2020). However, abnormal expression of FOX proteins cause cancer development, growth and distant metastasis (Yamashita et al. 2017; Wang et al. 2018). Despite the role of various FOX proteins were reported, the biological function and molecular mechanism of FOX proteins, such as FOXC1, remain in an early stage and need to be investigated further.

FOXC1 was first discovered in the mesenchyme of ocular drainage structures derive, causing abnormal ocular development (Smith et al. 2000). Tsutomu et al. discovered that FOXC1 contributed to the early organogenesis of the urinary trac and kidney (Kume et al. 2000). Observations by Ordan et al. presented that augmented FOXC1 expression resulted in the occurrence of glaucoma cataract and iris hypoplasia (Lehmann et al. 2000). The changing landscape of tumor biology reveals a growing body of proof indicating that FOXC1 is elevated in multiple types of malignant tumors, such as Lung cancer, triple-negative breast cancer, liver cancer, and gastrointestinal tumors (Han et al. 2018; Lin et al. 2021; Cao et al. 2018; Liu et al. 2018; Jiang et al. 2021), causing poor prognosis to these patients. Moreover, FOXC1 is recognized as a crucial regulator in the advancement of epithelial-mesenchymal transition (EMT), a critical mechanism implicated in the dissemination of cancer and resistance to drugs. (Diepenbruck and Christofori 2016). EMT is a phenotypical transformation within epithelial cells that lose the contacts of cell–cell basement membrane and their structural polarity become spindle-shaped (Singh and Settleman 2010). EMT markers change as cancer spreads (Schmalhofer et al. 2009). In cervical carcinoma, FOXC1 speeds up EMT progression via modulating PI3K/AKT pathway (Huang et al. 2017). In esophagus cancer, FOXC1 accelerates EMT progression by modifying ZEB2 expression (Zhu et al. 2017). In breast cancer, FOXC1 activates the transcription of FGFR1 to enhance the EMT progression (Hopkins et al. 2017). These studies suggest that FOXC1 is able to regulate EMT progression to promote cancer metastasis. FOXC1's biological function in RCC is unclear, however.

Our study revealed a reduction in FOXC1 expression in RCC tissues, leading to unsatisfactory prognosis for patients with RCC. Further experiments presented that FOXC1 inhibited RCC cell growth, metastasis and EMT progression. Next, we noticed that FOXC1 promoted the transcription of ABHD5 to regulating AMPK/mTOR pathway. Our study revealed a novel approach to fight against RCC via targeting FOXC1/ABHD5/ AMPK/mTOR pathway.

## Material and methods

### Tissue collection

RCC patients who underwent surgical treatment provided 64 RCC tissues and homologous adjacent cancer tissues. The Beijing Friendship Hospital's ethical committee reviewed the collection of RCC specimens. All patients included in our study agreed with our study and signed informed consent. All patients are aware their rights and responsibilities. Finally, the pathological diagnosis was determined by three experienced pathologists.

### Cell lines and transfection

For this study, American Type Culture Collection (ATCC) supplied human RCC cell lines (OS-RC-2, 769-P, ACHN, Caki-1 and 786-O), while HK2 cell was provided by Shanghai Academy of Biological Sciences. HK2 cells were grown in DMEM medium from Gibco in the United States, with the addition of antibiotics and fetal bovine serum from Israel BioIndustries. RCC cell lines, on the other hand, were grown in 1640 medium from Gibco in the United States. Those specific short hairpins (shRNAs) targeting on FOXC1 or ABHD5 was purchased from GenePharma (Suzhou, China). In order to boost gene expression, the coding region of FOXC1 or ABHD5 was incorporated into the pcDNA3.1 plasmid. Transfection of cells was carried out with the Lipofectamine 3000 kit (Invitrogen, USA) when cells reached a density of 50%-70%. This study used shRNA sequences could be found in Table S1.

### Cell proliferation assay

Cell growth was evaluated through three different tests. For CCK-8 assay, transfected RCC cells were digested and transferred into 96-well plates overnight. The spectrophotometer (Bio-Rad, USA) was utilized to evaluate the absorbance of each well in the plate at different time points. In the colony-formation test, around 1000 RCC cells that had been transfected were digested and then moved to a 6-well plate with sufficient medium, where they were cultured for a period of 14 days. Following fixation with paraformaldehyde, the cells were treated with crystal violet solution, washed with PBS solution, photographed, and then rinsed with glacial acetic acid to remove the colonies. EDU detection was performed using the EDU Proliferation Detection Kit (Guangzhou Ruibo Biotechnology). After being transfected, the cells were placed on a cell culture sheet in a 24-well plate overnight, followed by staining with EDU and DAPI fluorescent dyes. Finally, image of cells with fluorescence was captured by a microscope (leica, Germany).

### Cell metastasis

Migration capacity was assessed by scratch repair and Transwell migration assay. In the scratch repair assay, transfected cells were digested and transferred to a cell culture plate overnight. When the cells grow to a density of 90% to 100%, we utilized a pipette to draw a scar on the cell surface and washed away the necrotic cells with PBS solution. Cell images were taken at 0 h and 24 h using conventional light microscopy. During the Transwell migration assay, cells that had been transfected were grown in a medium without serum in the upper chamber. The transferred cells were treated with paraformaldehyde, then dyed using crystal violet solution, followed by imaging and dissolution in glacial acetic acid. Finally, we used a microplate reader to calculate the OD value of the washing solution at 550 nm. The process of Transwell invasion assay is almost the same as that of Transwell migration assay. The only difference is that Matrigel (Corning, USA) should be lay on the upper chamber in advance and then cells should be seeded.

### RT-qPCR assay

We washed RCC specimens and cells with PBS solution, added TRIzol reagent (ThermoFisher, USA) for lysis and extracted the samples with chloroform to purify RNA. The extracted RNA was stored in liquid nitrogen container. cDNA was transcribed by RNA mentioned above by utilizing a reverse transcription reagent (TAKARA, Japan). Toyobo Green RT-PCR Mix was utilized for RT-qPCR along with cDNA and primers that were custom-designed by our team. The experiment includes a list of all primers used, which can be found in Supplementary Table S2. The design of all primers was based on the coding sequence of each genes and using Primer-BLAST tool in the BLAST website. The overall capacity of the RT-qPCR reaction mixture was 10 μl, consisting of 1.4 μl distilled water, 5.0 μl RT-qPCR Master Mix, and 1.6 μl of primers (upstream and downstream primers account for half each) and 2.0 μl cDNA. The reaction was performed using the Roche LightCycler® 480II PCR system from Roche, Basel, Switzerland. The reaction parameters included at 95 °C for 30 s, at 95 °C for 5 s, at 55 °C for 10 s, at 72 °C for 15 s, repeated for 40 cycles. The annealing temperature varied between 55 °C and 65 °C. The relative expression of gene mRNA was determined by using the 2^−ΔΔCT^ computational formula.

### Western blot

After washing RCC specimens and cells with PBS solution, we added RIPA solution with protease inhibitor to purify protein and performed protein quantification. Equal amount of protein is added to a stacking gel. Electrophoresis is performed to separate proteins of different sizes, and then a PVDF membrane is used for protein transfer. The membrane was obstructed using 5% skim milk powder for one hour and rinsed thrice with TBST solution. After incubation with the corresponding primary antibodies, we washed membrane with TBST solution and added secondary antibodies. Finally, the treated membrane was visualized using the BioSpectrum 600 imaging system (UVP, USA).

### Immunohistochemistry

Formaldehyde solution was used to fix all tissue specimens in this study. All samples were dehydrated with different concentrations of alcohol, soaked in xylene solution twice, embedded by paraffin and cut. Paraffin samples were dewaxed with xylene solution and dehydrated with different concentrations of alcohol. Then, 3% H2O2 solution was applied to inactivate endogenous enzymes and sodium citrate-hydrochloric acid buffer solution was used for the recovery of antigen. Paraffin sections were covered with goat serumand then rinsed 3 times with TBST solution.

The main antibody was agitated and combined overnight, while the secondary antibody was agitated and combined at room temperature. Finally, the paraffin sections were stained with diaminobenzidine kit and imaged.

### Chromatin immunoprecipitation (CHIP) assay

ChIP kit (Bersinbio, Guangzhou) was conducted the CHIP assay. Briefly, 293 T cells transfected with FOXC1 overexpression vector grown to 80%-90% confluence of 10 cm plates. Next, cells were washed with PBS solution, treated with formaldehyde and then neutralized with glycine solution. The cells were exposed to a Lysis Buffer that included a protease inhibitor during incubation. Cell suspension were centrifuged and resuspended to collect nuclear pellet. Ultrasonic lysis was used to fragment the entire DNA, which was then attached to the FOXC1 antibody and left to incubate overnight at a temperature of 4 °C.The cell lysate was allowed to interact with protein A/G-beads at ambient temperature for half an hour. The bound protein-DNA complexes were collected by using magnetic frame and washed to perform cross-linkage reaction. Finally, DNA bound with FOXC1 protein were enriched and extracted for PCR and qRT-PCR assay. The primers for ABHD5 promoters are presented in listed in Table S3.

### Luciferase reporter assay

Forecasted binding sites of FOXC1 and ABHD5 were cloned into the pGL3 vector to conduct various reporter plasmids. Next, pcDNA3.1-FOXC1 and the reporter plasmid were simultaneously introduced into 293 T cells with the Lipofectamine 3000 kit, followed by measuring the luciferase activity of each sample using the Dual-Luciferase Reporter kit.

### In vivo assay

The animal experiments were conducted with nude mice, admitted and supervised by the Beijing Friendship Hospital ethics committee. 786-O cells that were stably expressing either the negative control, FOXC1, or FOXC1 + sh-ABHD5 were bred in larger numbers. We divided 15 5-week-old male nude mice into three groups using random sampling, including a group with overexpression of FOXC1, a group with FOXC1 and sh-ABHD5, and a negative control group. 786 cells that were stably expressing either the negative control, FOXC1, or FOXC1 + sh-ABHD5 were implanted into the subcutaneous tissue of nude mice. All xenograft tumors were measure by a graduated scale each week. Six weeks after injection, we euthanized all mice with carbon dioxide, completely peeled off the transplanted tumors from the epidermis, and weighed them. We then extracted RNA and proteins from the tumors and performed corresponding experiments. A lung metastatic tumor model was then established to verify RCC cell metastasis. 786-O cells stably expressing negative control, FOXC1, or FOXC1 + sh-ABHD5 were injected into the blood. Three weeks later, we euthanized all mice with carbon dioxid and in vivo imaging of nude mice lung metastasis mode were taken.

### Statistics for research

Statistics for research was conducted using SPSS 26.0 in Chicago, USA, with the differences between groups analyzed using nonparametric tests or independent-sample T analysis. Paired T analysis or non-parametric analysis was applied to analyze gene expression level in different tissues. A P value less than 0.05 signifies a notable distinction within our dataset.

## Results

### The level of FOXC1 was reduced in RCC samples

**Fig. 1:**
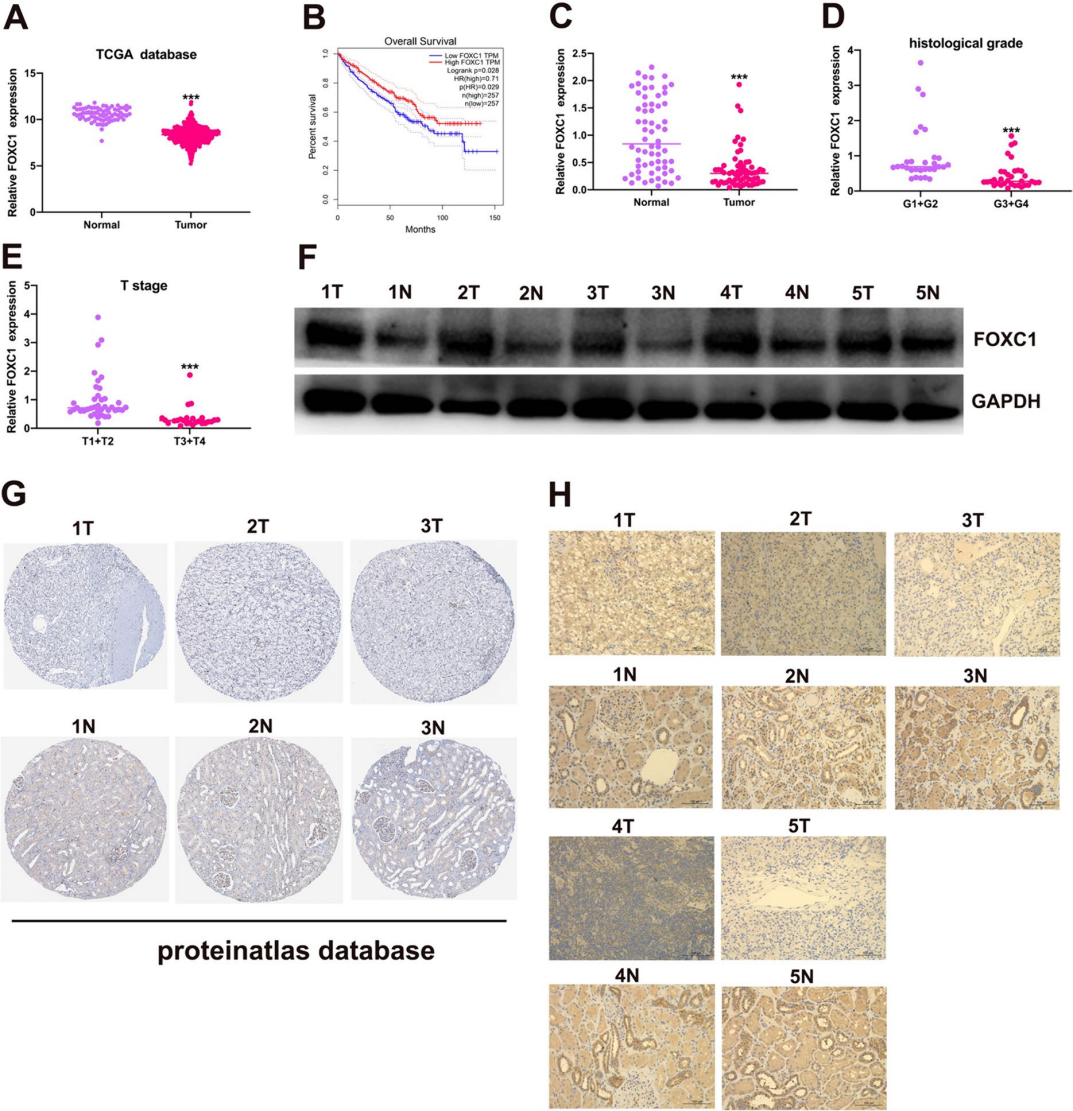
FOXC1 is down-regulated in RCC. (**A**) FOXC1 expression level in RCC tissues and normal tissues from TCGA database. (**B**) The overall survival of RCC patients with low or high FOXC1 expression from TCGA database. (**C**) FOXC1 expression level in RCC tissues and normal tissues from our hospital. (**D**) FOXC1 expression in RCC patients with different histological grade. (**E**) FOXC1 expression in RCC patients with different T stage. (**F**) FOXC1 protein expression level in 5 pairs of RCC tissues and match adjacent tissues. (**G**) IHC score of FOXC1 in RCC tissues and match adjacent tissues from proteinatlas database. (H) IHC score of FOXC1 in RCC tissues and match adjacent tissues from our hospitals. *p < 0.05, **p < 0.01, ***p < 0.001

Data from the TCGA database was utilized to compare FOXC1 mRNA expression levels in RCC tissues and normal tissues, revealing a significant decrease in FOXC1 mRNA expression in RCC tissues (Fig. 1A). Low FOXC1 expression led to unsatisfactory prognosis of these patients (Fig. 1B). Besides, FOXC1 expression was significantly decreased in RCC tissues collected from our hospital (Fig. 1C). Decreased FOXC1 mRNA expression has a relationship with histological grade (Fig. 1D) and T stage (Fig. 1E). Compared with matched adjacent adjacent tissues, FOXC1 protein expression was lower in 5 pairs of tumor tissues (Fig. 1F). The proteinatlas database and our hospitals both showed a significant decrease in FOXC1 expression in RCC tissues, suggesting a correlation between low FOXC1 expression and unfavorable outcomes for RCC patients.

### FOXC1 suppressed RCC cell proliferation

**Fig. 2:**
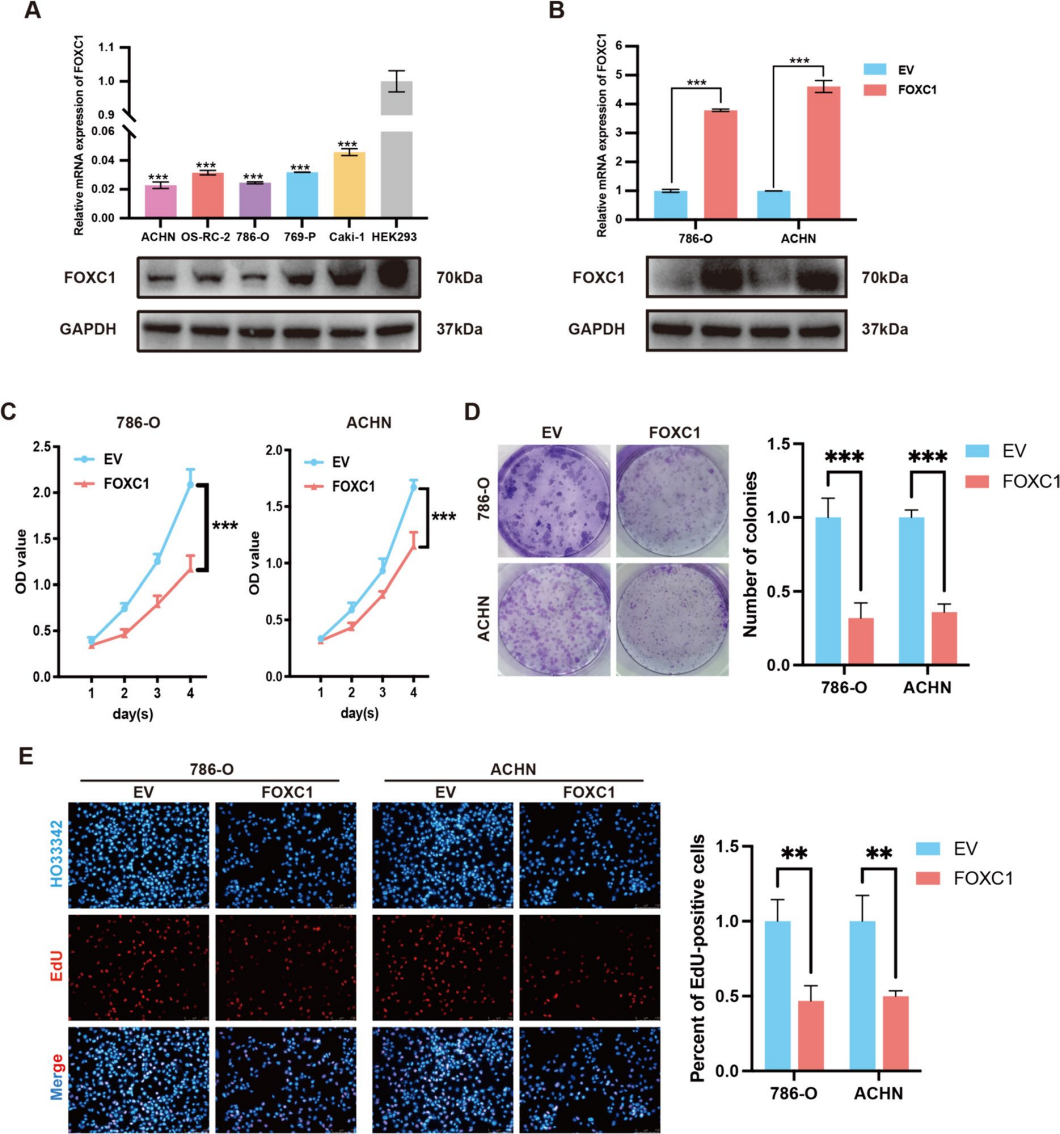
FOXC1 suppresses RCC proliferation in vitro. (**A**) FOXC1 mRNA and protein expression level in RCC cells and normal renal tubular epithelial cell. (**B**) qRT-PCR and western blot assay were applied to detect the expression level of FOXC1 while RCC cells were transfected with pcDNA3.1-FOXC1 or pcDNA3.1-NC. (**C**) CCK-8 assay showed the growth curves of RCC cells transfected with pcDNA3.1-FOXC1 or pcDNA3.1-NC. (**D**) Colony-formation assay was applied to evaluate RCC proliferation. (E) EDU assay was applied to evaluate RCC proliferation. *p < 0.05, **p < 0.01, ***p < 0.001

RT-qPCR and Western blotting techniques were used to evaluate FOXC1 expression in RCC cell lines and HK2 cells. A noticeable reduction in FOXC1 expression was observed in RCC cells, particularly in ACHN and 786-O cells (Fig. 2A). In order to enhance the inherent production of FOXC1, ACHN and 786-O cells were transfected with pcDNA3.1-FOXC1. (Fig. 2B). The ability of RCC cells to multiply was assessed through CCK-8, colony formation, and EdU tests.CCK-8 assay showed that increased levels of FOXC1 impeded the growth rate of RCC cells (Fig. 2C). Increased FOXC1 expression was found to significantly suppress the growth of RCC cells in colony formation and EdU assays (Fig. 2D and E).

### FOXC1 inhibited RCC cell metastasis via regulating EMT process

**Fig. 3:**
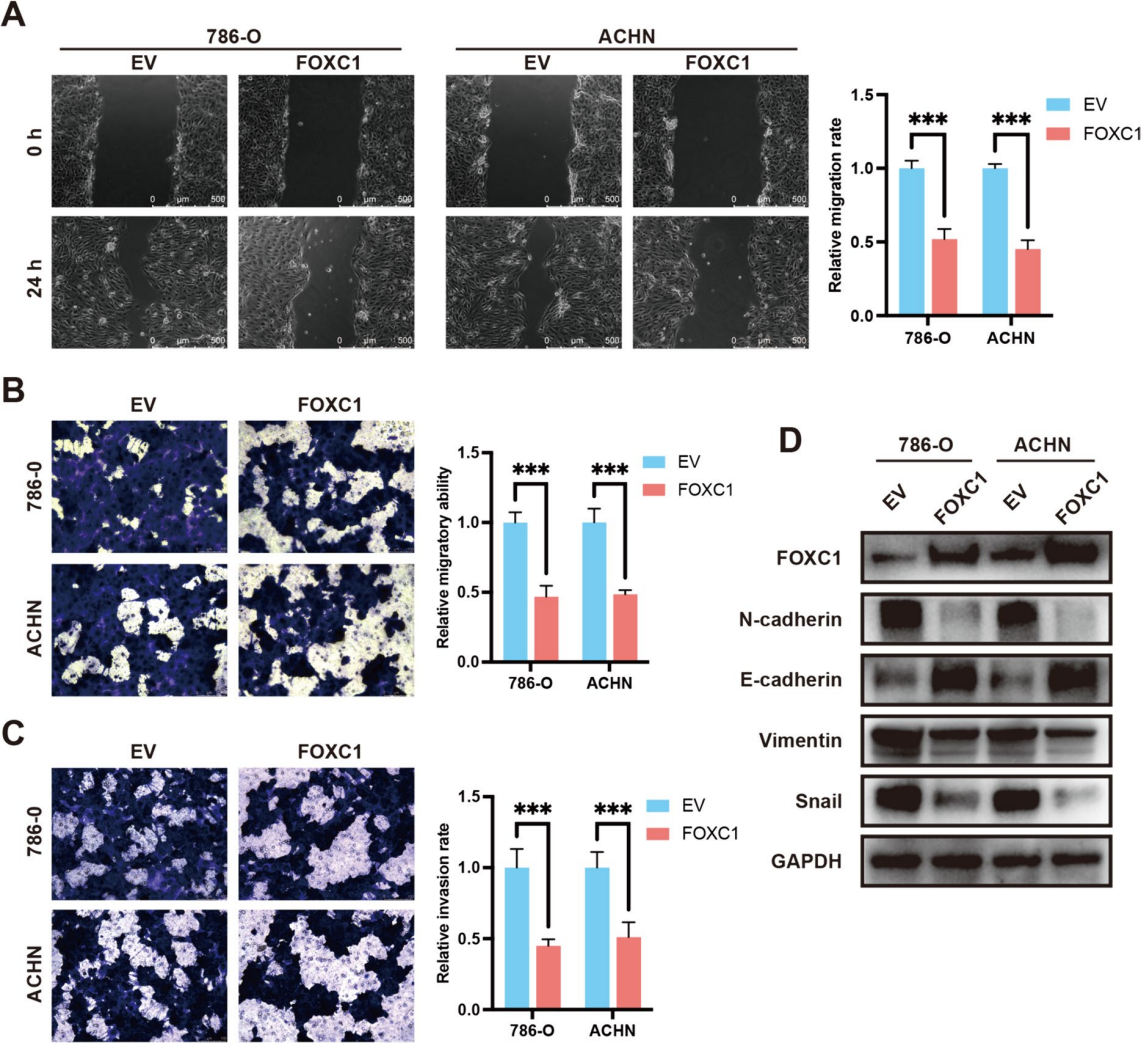
FOXC1 suppresses RCC migration, invasion and EMT process in vitro. (**A**) FOXC1 functions in RCC cell migration by using a wound healing assay. (**B**) FOXC1 functions in RCC cell migration by using a transwell migration assay. (**C**) FOXC1 functions in RCC cell invasion by using a transwell invasion assay. (**D**) FOXC1 functions in EMT-related proteins (Snail, Vimentin, N-cadherin and E-cadherin). *p < 0.05, **p < 0.01, ***p < 0.001

The migration ability of cell was evaluated by Transwell migration assay and scratch healing assay. The overexpression of FOXC1 clearly inhibited the migration capability of RCC, as shown in Fig. 3A and B). FOXC1 inhibits the cell invasive ability cells as shown by Transwell invasion assay (Fig. 3C). On the group of the role of FOXC1 on RCC cell metastasis, we supposed that FOXC1 could regulate RCC cell EMT progression. As shown in Fig. 3D, FOXC1 suppressed the levels of N-cadherin and Snail proteins, as well as Vimentin, while enhancing the expression of E-cadherin protein. Our results suggested that FOXC1 inhibited RCC cell metastasis and EMT progression.

### FOXC1 activates the transcription of ABHD5

**Fig. 4:**
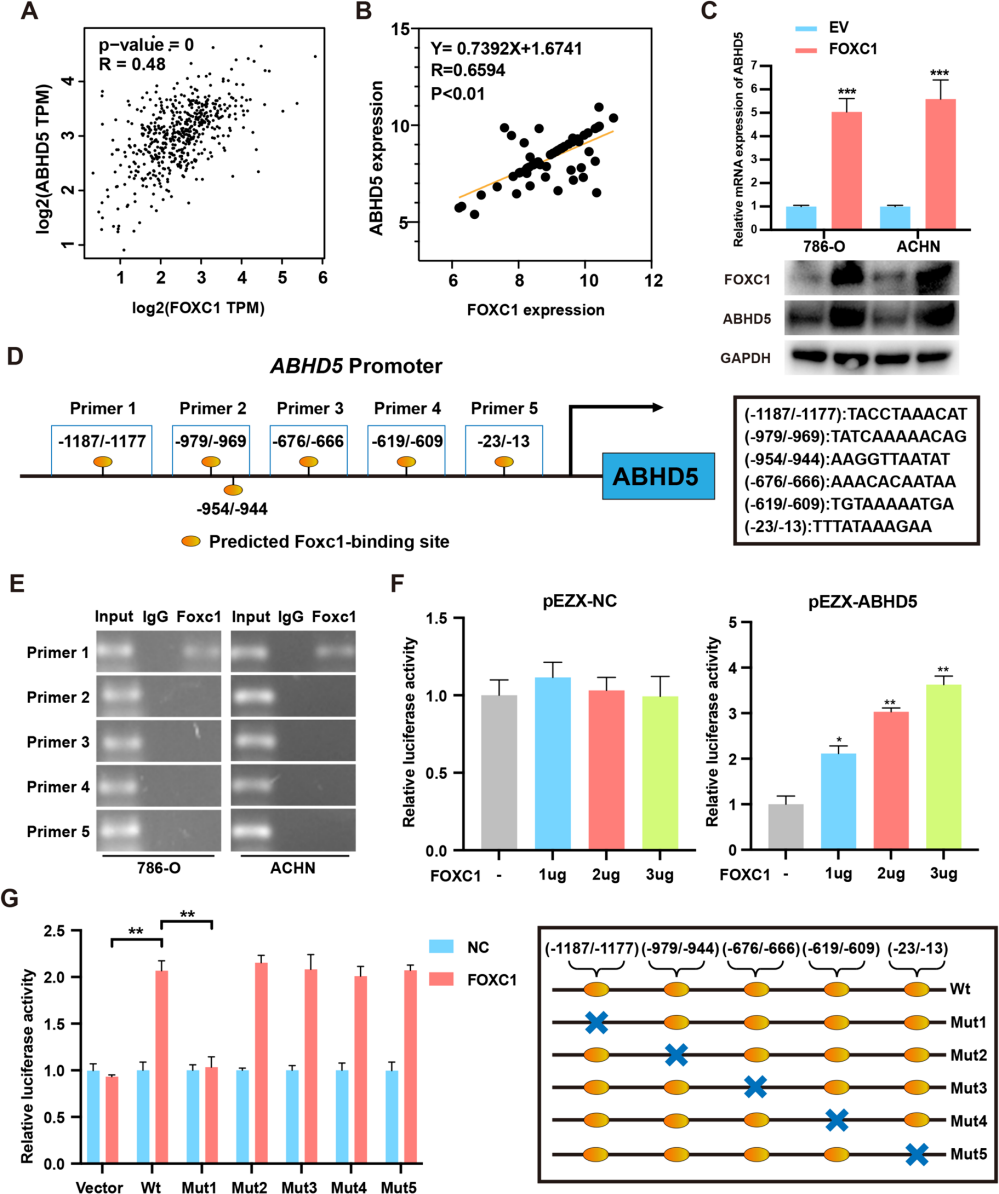
FOXC1 binds to ABHD5 promoter region and promotes ABHD5 transcription. (**A**) Correlation between FOXC1 and ABHD5 expression in TCGA database. (**B**) Correlation between FOXC1 and ABHD5 expression of RCC tissues in our hospitals. (**C**) qRT-PCR and western blot assay were applied to detect ABHD5 mRNA and protein level while RCC cells were transfected with pcDNA3.1-FOXC1 or pcDNA3.1-NC. (**D**) Six FOXC1-binding sties on the promoter of ABHD5 were forecasted thorough using JASPAR database. (**E**) CHIP and RT-PCR assay were conducted to investigate the direct binding sites of FOXC1 on ABHD5 promoter. (**F**) Luciferase assay were applied to evaluate the activity of 293 T cells transfected with ABHD5 promoter and different concentrations of FOXC1 plasmids. (**G**) The luciferase activities of 293 T cell co-transfected of WT or Mut ABHD5 promoter with FOXC1. *p < 0.05, **p < 0.01, ***p < 0.001

FOXC1 is a famous transcription factor, which can directly regulate the transcriptome. To identified the downstream genes of FOXC1, we detected 40 most relevant genes using the GEPIA database. Thirteen possible genes were discovered by analyzing the gene expression levels in healthy tissues and tissues affected by renal cell carcinoma (RCC). We mined the expression levels of these genes from the TCGA database. However, we discovered that overexpression of FOXC1 only enhanced ABHD5 expression dramatically (Supplemental Fig.S1). Moreover, FOXC1 mRNA expression has a positive correlation with ABHD5 mRNA expression in RCC tissues from TCGA database (Fig. 4A) and our samples (Fig. 4B). Increased FOXC1 expression led to higher levels of ABHD5 mRNA and protein (Fig. 4C). Next, we applied the JASPAR database to forecast ABHD5 common putative promoter sites binding with FOXC1 and discovered that six potential binding sites, including − 13/ − 23, − 609/ − 619, − 666/ − 676, − 944/ − 954, and − 969/ − 979 and − 1177/ − 1187 (Fig. 4D). CHIP and luciferase assay were carried out to identified above-mentioned binding sites. CHIP and RT-PCR assay demonstrated that FOXC1 bound the -1187 to -1177 region upstream of ABHD5 transcription start site (Fig. 4E). Luciferase assay suggested that forced expression of FOXC1 enhanced ABHD5 transcriptional activity via binding to the -1187 to -1177 region upstream of ABHD5 transcription start site (Fig. 4F). Finally, we mutated all these sites mentioned above and created five mutants (Mut1 to Mut5) in Fig. 4G and discovered that the luciferase activity was changed while co-transfection of KLF9 and Mut1 plasmid (Fig. 4G). These results indicate that FOXC1 promotes ABHD5 transcription via binding to -1187 to -1177 region upstream of ABHD5 transcription start site.

### The expression of ABHD5 was reduced in RCC tissues

**Fig. 5:**
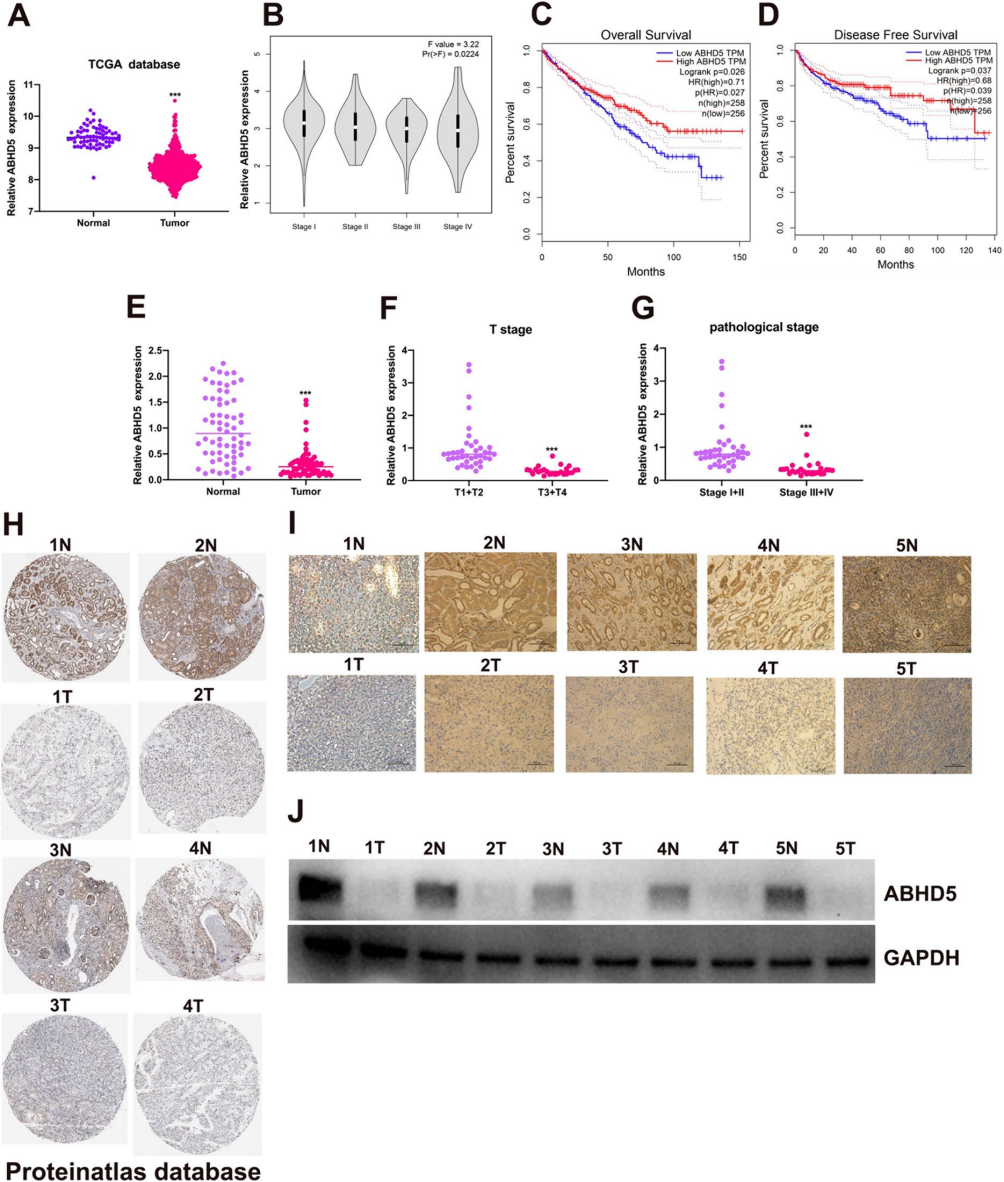
ABHD5 is down-regulated in RCC. (**A**) ABHD5 expression level in RCC tissues and normal tissues from TCGA database. (**B**) ABHD5 expression in RCC patients with different pathological stage. (**C**) The overall survival of RCC patients with low or high ABHD5 expression from TCGA database. (**D**) The disease-free survival of RCC patients with low or high ABHD5 expression from TCGA database. (**E**) ABHD5 expression level in RCC tissues and normal tissues from our hospitals. (F) ABHD5 expression in RCC patients with different T stage. (**G**) ABHD5 expression in RCC patients with different pathological stage. (**H**) IHC score of ABHD5 in RCC tissues and match adjacent tissues from proteinatlas database. (**I**) IHC score of ABHD5 in RCC tissues and match adjacent tissues from our hospitals. (**J**) ABHD5 protein expression level in 5 pairs of RCC tissues and match adjacent tissues. *p < 0.05, **p < 0.01, ***p < 0.001

By analyzing the data, the mRNA expression levels of ABHD5 in RCC tissues were compared with those in normal tissues in the TCGA database. The findings indicated a notable decrease in ABHD5 mRNA expression in RCC tissues. (Fig. 5A). Down-regulation of ABHD5 was positively with RCC pathological stage (Fig. 5B). Decreased ABHD5 expression in RCC patients caused unsatisfactory overall survival (Fig. 5C) and disease-free survival in these patients (Fig. 5D). We also tested RCC tissues in our hospital using qRT-PCR, and the test results showed that the expression of ABHD5 was significantly reduced. (Fig. 5E). Reduced ABHD5 mRNA levels were strongly linked to the pathological T stage (Fig. 5F) and pathological stage (Fig. 5G) in RCC. Furthermore, ABHD5 protein expression was obviously suppressed in RCC tissues from proteinatlas database (Fig. 5H) and our hospitals (Fig. 5I). Western blot assay also confirmed the results of immunohistochemistry (Fig. 5I). These results suggested that decreased ABHD5 expression caused unsatisfactory prognosis of RCC patients.

### The inhibition of cell growth and spread was achieved by ABHD5 through regulation of the AMPK/mTOR signaling pathway

**Fig. 6:**
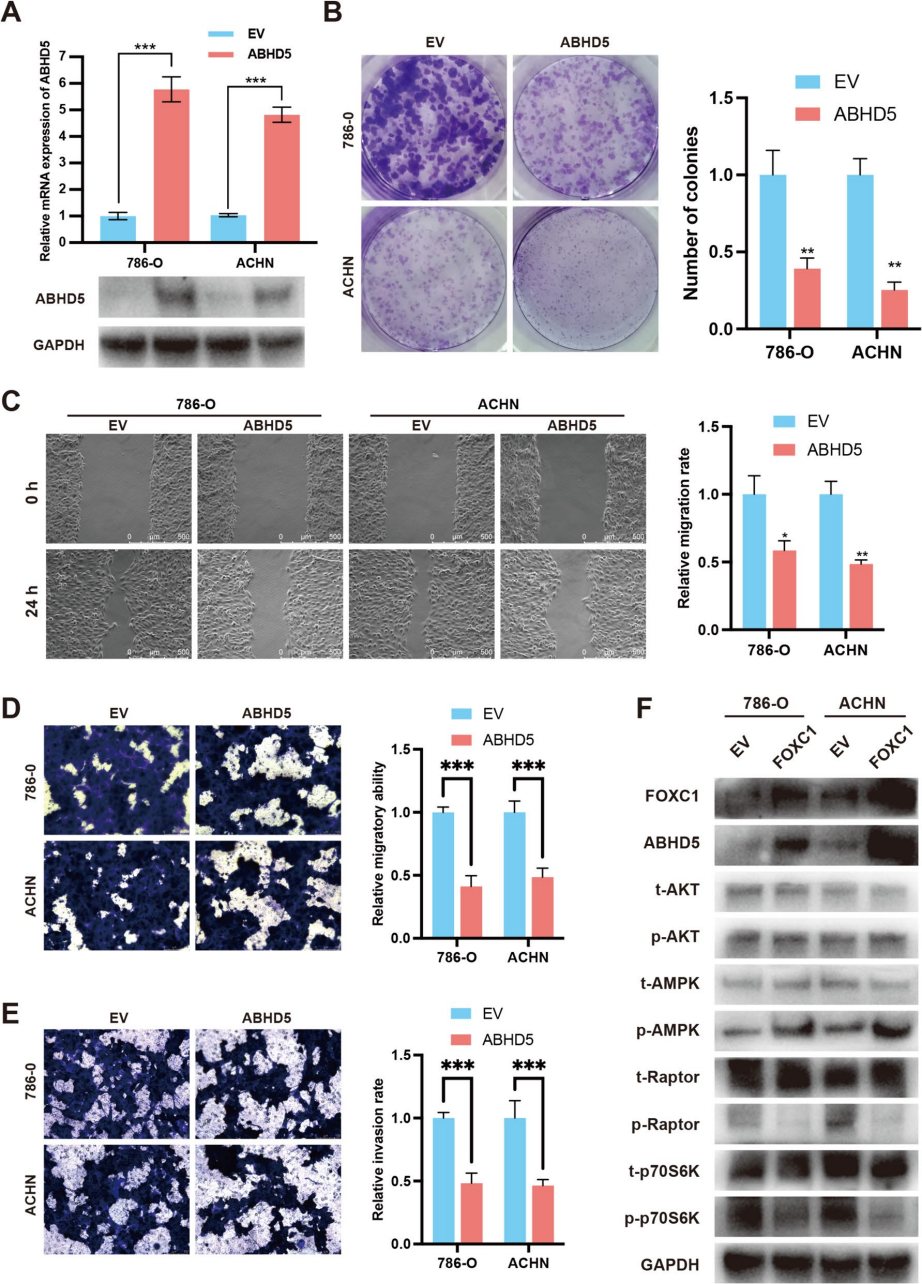
ABHD5 suppresses RCC proliferation and metastasis. (**A**) qRT-PCR and western blot assay were applied to detect the expression level of ABHD5 while RCC cells were transfected with pcDNA3.1-ABHD5 or pcDNA3.1-NC. (**B**) Colony-formation assay was applied to evaluate RCC proliferation. (**C**) Wound healing assay was applied to evaluate the migration of RCC cells transfected with pcDNA3.1-ABHD5 or pcDNA3.1-NC. (**D** and **E**) Transwell assay was applied to evaluate the migration and invasion of RCC cells transfected with pcDNA3.1-ABHD5 or pcDNA3.1-NC. (**F**) The activities of AMPK and mTOR signal pathway while RCC cells were transfected with pcDNA3.1-ABHD5 or pcDNA3.1-NC. *p < 0.05, **p < 0.01, ***p < 0.001

To boost the natural ABHD5 expression, pcDNA3.1-ABHD5 was integrated into 786-O and ACHN cells (Fig. 6A). The colon formation assay indicated that ABHD5 hinders the growth of RCC cells (Fig. 6B). Additionally, the scratch and transwell migration assays let us know that ABHD5 restricted the migration ability of RCC cells (Fig. 6C and D). Furthermore, the transwell invasion assay demonstrated that ABHD5 hindered the invasion ability of RCC cells (Fig. 6E). A previous study showed that ABHD5 activated the AMPK signaling pathway to suppress the mTOR signaling pathway, leading to the suppression of cancer cell anabolism (Chen et al. 2021). Subsequently, we used Western blotting assays to evaluate the activation of ABHD5-related signaling pathways. As shown in Fig. 6F, upregulation of FOXC1 increases ABHD5 protein level, thereby stimulating the AMPK signaling pathway but not the AKT signaling pathway. As expect, the activity of mTOR/P70SK6 signal pathway was suppressed upon AMPK signal pathway activation (Fig. 6F). These results indicate that FOXC1 promotes ABHD5 transcription to regulate AMPK/mTOR signal pathway, thereby inhibiting RCC cell proliferation and metastasis.

### Knockdown of ABHD5 restores cell growth and metastasis suppression caused by FOXC1 enhancement

**Fig. 7:**
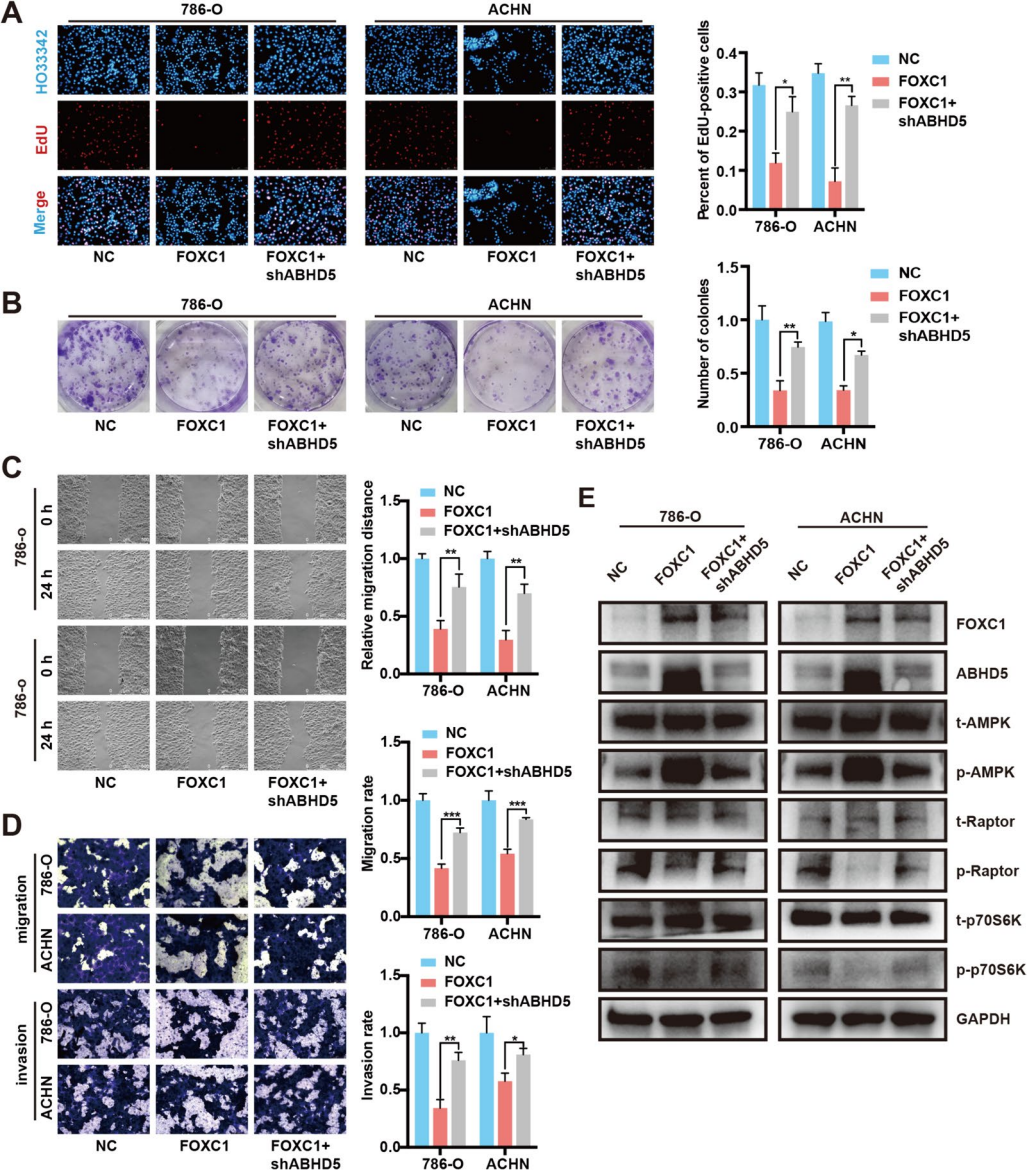
Suppression of ABHD5 reverses the inhibitory effect of FOXC1 overexpression on cell growth and metastasis. (**A**) EDU assay was applied to detect the proliferation of RCC cells transfected with pcDNA3.1-NC, pcDNA3.1-FOXC1 or pcDNA3.1-FOXC1 + shABHD5. (**B**) Colony-formation assay was used to investigate the proliferation of RCC cells transfected with pcDNA3.1-NC, pcDNA3.1-FOXC1 or pcDNA3.1-FOXC1 + shABHD5. (**C**) Wound healing assay was employed to investigate the proliferation of RCC cells transfected with pcDNA3.1-NC, pcDNA3.1-FOXC1 or pcDNA3.1-FOXC1 + shABHD5. (**D**) Transwell assay was applied to evaluate the migration and invasion of RCC cells transfected with pcDNA3.1-NC, pcDNA3.1-FOXC1 or pcDNA3.1-FOXC1 + shABHD5. (**E**) The activities of AMPK and mTOR signal pathway while RCC cells were transfected with pcDNA3.1-NC, pcDNA3.1-FOXC1 or pcDNA3.1-FOXC1 + shABHD5. *p < 0.05, **p < 0.01, ***p < 0.001

To investigate whether FOXC1 functions by mediating the expression of ABHD5, a rescue assay among FOXC1 and ABHD5 was conducted. EdU and Colony-formation experiments presented that suppression of ABHD5 significantly regained RCC cell growth inhibition mediated by FOXC1 overexpression (Fig. 7A and B).In RCC cells, the inhibition of ABHD5 reversed the migration and invasion suppression caused by overexpression of FOXC1, as demonstrated by scratch test and transwell experiment (Fig. 7C and D).Finally, western blot assay showed that overexpression of FOXC1 activated AMPK signal pathway to suppress mTOR signal pathway, whereas suppression of ABHD5 reversed these effects (Fig. 7E). In vitro, it is possible to reverse the suppression of cell growth and metastasis induced by FOXC1 overexpression by simultaneously reducing ABHD5 levels, as suggested by these results.

### Overexpression of FOXC1 suppressed cell proliferation and metastasis of RCC in nude mice model

**Fig. 8:**
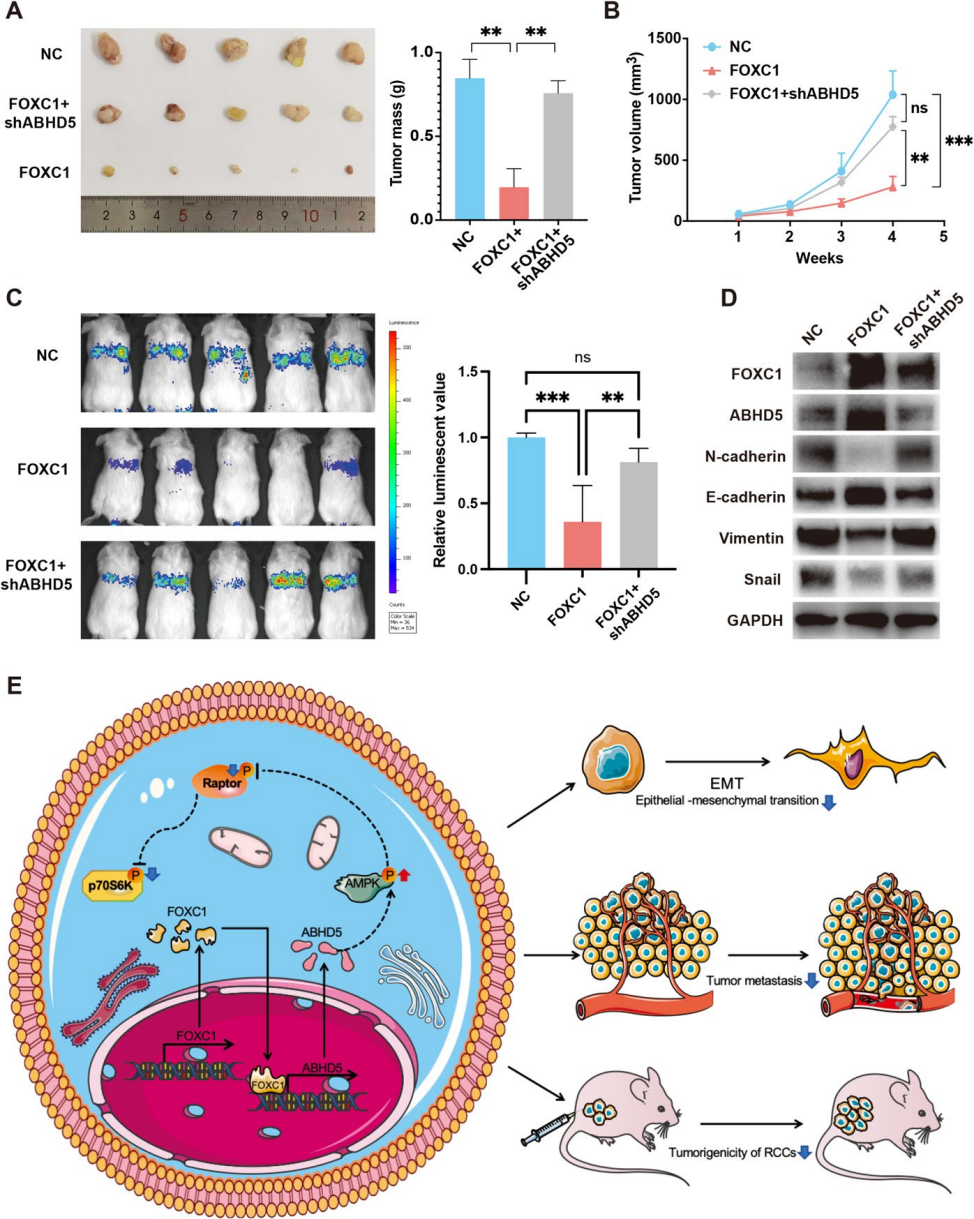
FOXC1 inhibited cell growth and metastasis of RCC cells in vivo. (**A**) The weight of xenograft tumor derived from 786-O cells stably expressing NC, FOXC1 or FOXC1 + sh-ABHD5. (**B**) The volume of xenograft tumor derived from 786-O cells stably expressing NC, FOXC1 or FOXC1 + sh-ABHD5. (**C**) The luciferase activities of nude mice injected with 786-O cells stably expressing NC, FOXC1 or FOXC1 + sh-ABHD5. (**D**) The protein expression level of FOXC1, ABHD5, E-cadherin, N-cadherin, Snail and Vimentin in xenograft tumor derived from 786-O cells stably expressing NC, FOXC1 or FOXC1 + sh-ABHD5. (**E**) Schematic diagram displaying the mechanism underlying FOXC1/ABHD5/AMPK/mTOR in RCC progression. *p < 0.05, **p < 0.01, ***p < 0.001

786-O cells were implanted into nude mice subcutaneously to create a xenograft tumor model or induce lung metastasis by injecting into the caudal vein, with stable expression of negative control, FOXC1, or FOXC1 + sh-ABHD5.Overexpression of FOXC1 decreased RCC cell growth ability in vivo, whereas suppression of ABHD5 reversed this effect (Fig. 8A and B). In addition, in vivo imaging of nude mice lung metastasis mode demonstrated that Overexpression of FOXC1 inhibited RCC cell metastasis in vivo, whereas suppression of ABHD5 reversed the inhibitory effect caused by FOXC1 overexpression (Fig. 8C). Subsequently, protein of subcutaneous tumors for western blot results presented that FOXC1 inhibited the expression of N-cadherin, Vimentin, and Snail while promoting E-cadherin levels. Conversely, inhibiting ABHD5 reversed these outcomes (Fig. 8D). In vivo, it is possible to reverse the inhibition of cell growth and metastasis resulting from FOXC1 overexpression by simultaneously reducing ABHD5 levels, as suggested by these results.

## Discussion

RCC is a fatal urologic tumor, bringing immense suffering and financial burden to these patients and society. Despite years of struggle in surgery, targeted therapy and immunotherapy, no significant progress has been made and the molecular mechanisms underlying RCC development and metastasis remains mysterious. Here, we observed that FOXC1 expression was dramatically reduced in RCC tissues, resulting unsatisfactory of prognostic to these patients. Further experiments demonstrated that FOXC1 acted as a tumor suppressor to regulating AMPK/mTOR pathway (Fig. 8E).

FOXC1 is located in chromosome 6p25 and also named as FREAC3 or FKHL7, which can encode a functional protein with 553 amino (Larsson et al. 1995; Kume et al. 1998). FOXC1 protein contains four main domains, including a forkhead DNA-binding domain (FHD) and a transcriptional activation domain situated at N-terminal (TAD-1), another TAD located at C-terminal (TAD-2) and a transcription inhibitory/phosphorylation domain (TIPD) (Han et al. 2017). TAD-1 and TAD-2 are positioned at 1–51 and 466–553, respectively. FHD is located at the 69–178 amino acid, while TIPD is located at the 215–366 amino acid (Berry et al. 2002; Yang et al. 2024). Besides the phosphorylation regulation, FOXC1 transcriptional activity is also modified by small ubiquitin-like modifier (Danciu et al. 2012). Research has demonstrated that FOXC1 is disrupted in various cancer, functioning as an oncogene in the advancement of the majority of cancer types. In breast cancer, abnormally expressed FOXC1 can activate downstream pathways by enhancing NF-KB transcription, thereby facilitating cell growth and metastasis (Wang et al. 2012). In triple-negative breast cancer, FOXC1 can increase cell metastasis by stimulating the transcription of CXC chemokine receptor-4 (CXCR4) (Pan et al. 2018). In gastric cancer, FOXC1 promotes cell proliferation by enhancing GPX8 transcription to activate Wnt signaling pathway (Chen et al. 2020). FOXC1 enhances cell growth and reduces lactate generation and glucose usage in colorectal cancer by suppressing FBP1 expression (Li et al. 2019). During the occurrence of lung cancer, abnormally expressed FOXC1 can activate corresponding signaling pathways by promoting lysyl oxygen (LOX) transcription, thereby accelerating tumor metastasis (Gong et al. 2019). However, we found that the expression of FOXC1 was significantly decreased in the tissues and cell lines of RCC. The tissue-specific expressions of genes related to cell division are biologically significant. Many genes related to cell growth are changed during the development of cancer due to various contributing factors (Sack et al. 2018). Furthermore, the study found that upregulation of FOXC1 significantly reduced the proliferation and spread of RCC both in vivo and in laboratory settings. However, the downstream target gene regulated by FOXC1 in RCC remains unclear. FOXC1, a well-known transcription factor, can increase the expression of tumor suppressor genes to demonstrate its anti-cancer properties. GEPIA database was employed to investigate 40 genes associated with FOXC1.Bioinformatics analysis was conducted to eliminate the oncogenes in RCC. Subsequently, a RT-qPCR test was conducted to confirm the regulation of ABHD5 mRNA expression by FOXC1.Analysis of ABHD5 promoter region by using JASPAR database suggested six putative FOXC1 binding sites. Luciferase reporter assay, CHIP assay and RT-qRCR assay confirmed that FOXC1 binds to the -1187 to -1177 region upstream of ABHD5 transcription start site. Nevertheless, the binding sites or domains in FOXC1 remains unclear. Moreover, the transcriptional cofactor associated with FOXC1 has not yet reported now. Finally, the component of transcription factor complex associated with FOXC1 is needed to be clarified. ABHD5 has been investigated in various research for its role in various forms of cancer. In colon cancer, ABHD5 impairs the progression of EMT and accelerates aerobic glycolysis via enhancing the activity of AMPKα-p53 pathway (Ou et al. 2014). Moreover, ABHD5 antagonizes the binding of CASP3 to the BECN1 cleavage site and prevents CASP3 from cleavage of BECN1, thereby accelerating autophagy-dependent cell death (Peng et al. 2016). Gu et al. discovers that ABHD5 inhibited the stemness of colon cancer cells via impairing DPY30 nuclear translocation and SET1A activity (Gu et al. 2021). In prostate cancer, ABHD5 impairs cell proliferation, metastasis and EMT progression via facilitating aerobic glycolysis and inhibiting mitochondrial respiration (Chen et al. 2017). Chen et al. observed that ABHD5 suppressed cell growth and caused cell cycle arrest by activating AMPK signal pathway and suppressing mTORC1 signal pathway (Chen et al. 2021). Here, we also discover that ABHD5 activate AMPK signal pathway instead of AKT signal pathway, thus suppressing mTOR pathway. AMPK functions as a detector of cellular metabolic status, becoming active when intracellular ATP levels are low due to various stresses (Shaw 2009). Multiple studies have shown that the AMPK signaling pathway significantly affects the key downstream mTOR pathway (Chomanicova et al. 2021; Wang and Guan 2009; Green et al. 2011). mTOR has two functionally complexes, including mTORC1 and mTORC2.mTORC1 consists of Raptor, PRAS40, DEPTOR, mLST8, and TSC1/TSC2 complexes, whereas mTORC2 is made up of Rictor, TSC1/TSC2, DEPTOR, and mLST8 complexes (Kim et al. 2017; Tamaddoni et al. 2020). p70S6K1, ULK1, and 4EBP1 are the targets of mTORC1, while PKC, Akt, and SGK are the targets of mTORC2 (Beevers et al. 2009). AMPK directly phosphorylates TSC2 at Ser1345 to activate its activity, thus activates the activity of mTORC1 (Gwinn et al. 2008). In addition, Akt indirectly phosphorylates TSC2 at S939 and T1462 to activate its activity, thus activates the activity of mTORC1 (Inoki et al. 2002; Manning et al. 2002). Akt also directly phosphorylates PRAS40 to impair mTORC1 activity and directly phosphorylate mTOR at S2448 to activate mTOR activity (Sancak et al. 2007; Nave et al. 1999). In this research, we discovered that FOXC1 increase the expression of ABHD5 to activate AMPK pathway instead of Akt pathway, thus inhibit the activity of mTOR pathway. Rescue assays ultimately showed that inhibiting ABHD5 can counteract the suppressive impact of FOXC1 overexpression on cell proliferation and metastasis. Overexpression of FOXC1 activated AMPK signal pathway to suppress mTOR signal pathway, whereas suppression of ABHD5 reversed these effects.

In summary, we have determined that FOXC1 functions as a cancer inhibitor in RCC and is linked to unfavorable prognosis. FOXC1 regulates the AMPK/mTOR pathway by enhancing ABHD5 transcription, thereby inhibitor RCC growth and metastasis. According to our study, FOXC1 could be used as a possible indicator for predicting the outcome of RCC and directing treatment. The signal pathway FOXC1/ABHD5/AMPK/mTOR presents new insight in RCC pathogenesis and metastasis.

## Supplementary Information

Below is the link to the electronic supplementary material.

Supplementary file1 (DOCX 1319 KB)

Supplementary file2 (DOCX 17 KB)

## Data Availability

The data used to support the findings of this study is available from the corresponding authors upon request.
