# Supplementary material for: FOXC1 transcriptionally suppresses ABHD5 to inhibit the progression of renal cell carcinoma through AMPK/mTOR pathway
Source: Cell Biol Toxicol. 2024 Aug 2;40(1):62. doi: 10.1007/s10565-024-09899-w (PMC11297099; doi:10.1007/s10565-024-09899-w)
Supplement: Supplementary file 1 — Supplementary file1 (DOCX 1319 KB) [file 10565_2024_9899_MOESM1_ESM.docx]

Supplementary Fig S1. The expression level of target genes in ACHN cells overexpressing FOXC1. (A-L) The expression level of BAMBI, CCDC160, CROT, FLRT3, GMD3, NAPEPLD, NT5C2, PSMD6, RLN2, SAMD13, SERAC1, WDR48 in ACHN cells transfected with pcDNA3.1-FOXC1 or pcDNA3.1-NC. (M) The expression level of ABHD5 in 786-O and ACHN cells overexpressing FOXC1. NS: no significant, *p < 0.05, **p < 0.01, ***p < 0.001.
