# Supplementary material for: FOXC1 transcriptionally suppresses ABHD5 to inhibit the progression of renal cell carcinoma through AMPK/mTOR pathway
Source: Cell Biol Toxicol. 2024 Aug 2;40(1):62. doi: 10.1007/s10565-024-09899-w (PMC11297099; doi:10.1007/s10565-024-09899-w)
Supplement: Supplementary file 2 — Supplementary file2 (DOCX 17 KB) [file 10565_2024_9899_MOESM2_ESM.docx]

Table S1. The oligonucleotide sequence of shRNAs used in our study.

| Primer name | Sequence (5’-3’) |
| --- | --- |
| shRNA-ABHD5-1 | GGAGATCTTTGCACCAACAGA |
| shRNA-ABHD5-2 | GGCCTGATTTCAAACGAAAGT |
| shRNA-NC | TTCTCCGAACGTGTCACGT |

Table S2. The sequence of primers used in qRT-PCR assay.

| gene | Sequence (5’-3’) |
| --- | --- |
| SAMD13-F | AGTAAAGGAACCCTGCAGCC |
| SAMD13-R | AGATTTTCAGAGCAGGCCCC |
| [FLRT3](http://gepia.cancer-pku.cn/detail.php?gene=FLRT3)-F | TGCGATGCGGGTTTCATTTACTG |
| [FLRT3](http://gepia.cancer-pku.cn/detail.php?gene=FLRT3)-R | ACATACTTTGGGAGGTTGGTAGGA |
| [NAPEPLD](http://gepia.cancer-pku.cn/detail.php?gene=NAPEPLD)-F | GGGAGATTTGTGAATCCGTGGC |
| [NAPEPLD](http://gepia.cancer-pku.cn/detail.php?gene=NAPEPLD)-R | CACCATTACCGTGGCATGTCC |
| [GMDS](http://gepia.cancer-pku.cn/detail.php?gene=GMDS)-F | GGAATTGTACGGCGGTCCAGTT |
| [GMDS](http://gepia.cancer-pku.cn/detail.php?gene=GMDS)-R | GCAGTGTACTCAGCGAGGTCAA |
| [RLN2](http://gepia.cancer-pku.cn/detail.php?gene=RLN2)-F | TGCTAATTTGCCACAGGAGCTG |
| [RLN2](http://gepia.cancer-pku.cn/detail.php?gene=RLN2)-R | ACTGCTGTCTGCGGCTTCAC |
| [CROT](http://gepia.cancer-pku.cn/detail.php?gene=CROT)-F | ATTGGCTGGAAGAGTGGTGG |
| [CROT](http://gepia.cancer-pku.cn/detail.php?gene=CROT)-R | AGCTGCCAGTAGTTCAAGTT |
| [WDR48](http://gepia.cancer-pku.cn/detail.php?gene=WDR48)-F | CAGCTGGGTTGGACAGACAA |
| [WDR48](http://gepia.cancer-pku.cn/detail.php?gene=WDR48)-R | GGATCCCATACCCGTAACACC |
| [SERAC1](http://gepia.cancer-pku.cn/detail.php?gene=SERAC1)-F | GCCCTGAAGAAGGCTGTGACA |
| [SERAC1](http://gepia.cancer-pku.cn/detail.php?gene=SERAC1)-R | TCCGCAGTATCTTGGCTGATGT |
| [BAMBI](http://gepia.cancer-pku.cn/detail.php?gene=BAMBI)-F | CTACTGTGATGCTGCCCACT |
| [BAMBI](http://gepia.cancer-pku.cn/detail.php?gene=BAMBI)-R | CATCGTGCAGCCCTCTGTAA |
| [PSMD6](http://gepia.cancer-pku.cn/detail.php?gene=PSMD6)-F | ACAGAATCCGGCAAGAGCAA |
| [PSMD6](http://gepia.cancer-pku.cn/detail.php?gene=PSMD6)-R | TGTAGGAAGGCATGCGATGG |
| CCDC160-F | ACTGCAGATAGCAGCAAGGG |
| CCDC160-R | AGGATGCAGAATTTGTGTCTGT |
| SOSTDC1-F | ACATCTCTGATGGCCAGTGC |
| SOSTDC1-R | TCATTGACACACCGCCACTC |
| [NT5C2](http://gepia.cancer-pku.cn/detail.php?gene=NT5C2)-F | ATCGGGTGTTTGTGAACCGA |
| [NT5C2](http://gepia.cancer-pku.cn/detail.php?gene=NT5C2)-R | GAGCAACTCCTGGGGATAGC |
| ABHD5-F | CCGGCTTCGAGATAAGTCCC |
| ABHD5-R | GCCAACCAGTTAGCCATCCT |
| FOXC1-F | TAAGCCCATGAATCAGCCG |
| FOXC1-R | GCCGCACAGTCCCATCTCT |
| GAPDH-F | GAGTCAACGGATTTGGTCGT |
| GAPDH-R | GACAAGCTTCCCGTTCTCAG |

Table S3. The oligonucleotide sequence of primers used in CHIP assay.

| Primer name | Sequence (5’-3’) |
| --- | --- |
| Primer 5-F | AAGAATTACCTGGGACTGG |
| Primer 5-R | TCTCTTGCTCCTGCTCTA |
| Primer 4-F | CTTTAGTTTCTCAGCTCCTTC |
| Primer 4-R | TTTAGGGAAGACCAGAACAT |
| Primer 3-F | ATGTTCTGGTCTTCCCTAAA |
| Primer 3-R | AGGAATGGTGTCTTCTGTAA |
| Primer 2-F | CAGGTTTACAGTGGGGCTCA |
| Primer 2-R | AGGTGCTTGAAAACAATGGGT |
| Primer 1-F | ACAGGTCAAGAAATACTGCT |
| Primer 1-R | GGGCAACAAGAACGAAAC |
